# Complete Genome Sequences of Three Staphylococcus haemolyticus Strains Isolated from the Lung of a TGFβ1 Transgenic Mouse with Lung Fibrosis

**DOI:** 10.1128/mra.01176-21

**Published:** 2022-03-07

**Authors:** Ahmed M. Abdel-Hamid, Corina N. D’Alessandro-Gabazza, Taro Yasuma, Kimberly K. O. Walden, Christopher J. Fields, Esteban C. Gabazza, Isaac Cann

**Affiliations:** a Carl R. Woese Institute for Genomic Biology (Microbiome Metabolic Engineering), University of Illinois at Urbana-Champaign, Urbana, Illinois, USA; b Department of Botany and Microbiology, Faculty of Science, Minia University, El-Minia, Egypt; c Department of Immunology, Mie University Faculty and Graduate School of Medicine, Tsu, Mie, Japan; d Center for Intractable Diseases, Mie University, Tsu, Mie, Japan; e Roy J. Carver Biotechnology Center, University of Illinois at Urbana-Champaign, Urbana, Illinois, USA; f Department of Animal Science, University of Illinois at Urbana-Champaign, Urbana, Illinois, USA; g Department of Microbiology, University of Illinois at Urbana-Champaign, Urbana, Illinois, USA; h Division of Nutritional Sciences, University of Illinois at Urbana-Champaign, Urbana, Illinois, USA; i Center for East Asian & Pacific Studies, University of Illinois at Urbana-Champaign, Urbana, Illinois, USA; University of Rochester School of Medicine and Dentistry

## Abstract

We report here the complete genome sequences of three Staphylococcus haemolyticus strains isolated from a mouse fibrotic lung tissue and exhibiting proapoptotic activity on human lung alveolar epithelial cells. The genomes were obtained from a combination of Illumina MiSeq and Oxford Nanopore MinION sequencing.

## ANNOUNCEMENT

Idiopathic pulmonary fibrosis (IPF) is a disease of unknown etiology, and the lung microbiome of IPF patients is characterized by increases in Staphylococcus and Streptococcus genera (1). We demonstrated that corisin, a peptide in diverse staphylococci, induces apoptosis of lung alveolar epithelial cells, a hallmark of IPF (2). Tissues from IPF patients and from transforming growth factor β1 (TGFβ1) transgenic (TG) mice with lung fibrosis enrich for halophilic bacteria (3). To isolate proapoptotic bacteria associated with IPF, tissue samples from the fibrotic lung of TGFβ1 TG mice (2) were inoculated into the high-salt ATCC medium 1097 and incubated at 37°C with shaking (220 rpm) until growth was visible. Bacterial colonies were obtained by plating the enriched culture on ATCC 1097 agar plates. One colony was further purified by streaking, and three colonies designated 1b, 7b, and 12b were picked, demonstrated to be proapoptotic, and subjected to genome sequencing. After growth in ATCC 1097 liquid medium for 24 h, genomic DNA was extracted using Qiagen Genomic-tip 20/g. Genome sequencing was then carried out by the Carver Biotech Center (UIUC) using paired-end short-read Illumina MiSeq and long-read MinION technologies (ONT). The shotgun genomic libraries, prepared with the HyperPrep Library construction kit from Kapa Biosystems (Roche), were pooled, quantitated, and sequenced on one MiSeq nano flow cell using a MiSeq 500-cycle sequencing kit (v2). Fastq files were demultiplexed with the bcl2fastq conversion software (Illumina; v2.20), and adaptors were trimmed from the 3′ ends of the reads. For ONT long-read sequencing, unsheared genomic DNA (1 μg) was converted into a barcoded Nanopore library with the NBD-114 and 1D library kit SQK-LSK109 from ONT and pooled for sequencing on a SpotON R9.4.1 FLO-MIN106 flow cell (GridIon X5 sequencer).

Illumina MiSeq reads and ONT long reads were checked for quality prior to and after trimming using FastQC (v0.11.8). MiSeq reads were trimmed using Trimmomatic v0.38, retaining reads longer than 30 bp (4). Long reads were adapter trimmed with Porechop (v0.2.3) and length filtered to a minimum of 1 kb with seqtk (v1.3). Unicycler v0.4.8 assembled the trimmed MiSeq and uncorrected ONT reads in a hybrid assembly using the default “normal” mode (5). Within Unicycler, the MiSeq reads were assembled with SPAdes (v3.11.1) (6), and the resulting long-anchor contigs were assembled together with the ONT reads by miniasm (7) and Racon (v0.5.0) (8). Pilon (v1.23) was used within Unicycler to iteratively polish the assembly with the MiSeq reads (9). Assemblies were evaluated for completeness using BUSCO (v3.0.1) (10) and QUAST (11) with GCF_002954055.1 (Staphylococcus epidermidis) as a reference. The sequence reads from each of strains 1b and 7b were assembled by Unicycler as a single circular chromosome and three circular plasmids (Bandage assembly graph viewer) (12). The reads from strain 12b were assembled as a single circular chromosome and two circular plasmids. The circularized chromosomes and plasmids were reoriented automatically by the Unicycler assembler to start with the replication initiation proteins DnaA and RepA, respectively. The genome assemblies were annotated using the NCBI Prokaryotic Genome Annotation Pipeline (PGAP) (13). The 16S rRNA genes identified the bacteria as Staphylococcus haemolyticus, and they were designated strains 1b, 7b, and 12b, respectively. Detailed information for the genomes is provided in Table 1.

**TABLE 1:** Genome characteristic of three of Staphylococcus haemolyticus strains isolated from lungs of TGFβ1 TG mice with lung fibrosis

Characteristic	Data for Staphylococcus haemolyticus strain:		
	1b	7b	12b
Whole-genome characteristics			
BioSample accession no.	SAMN18212548	SAMN18212549	SAMN18212550
Total no. of reads (Illumina)	173,026	138,020	146,291
SRA accession no. (Illumina)	SRX13955341	SRX13955342	SRX13955343
Total no. of reads (ONT)	136,577	243,795	256,825
SRA accession no. (ONT)	SRX13955344	SRX13955345	SRX13955346
Oxford Nanopore *N*_50_ (bp)	11,643	11,730	11,624
Genome size (no. of bp)	2,390,652	2,398,463	2,382,565
GC content (%)	32.83	32.76	32.76
Total no. of genes	2,373	2,382	2,364
No. of chromosomes + plasmids	1 + 3	1 + 3	1 + 2
*N*_50_ (bp)	2,339,731	2,341,142	2,339,810
Chromosome			
GenBank accession no.	CP071512	CP071508	CP071505
Illumina short-read coverage	30×	30×	36×
Chromosome size (bp)	2,339,731	2,341,142	2,339,810
Chromosome topology	Circular	Circular	Circular
Total no. of chromosomal genes	2,317	2,319	2,316
No. of proteins	2,132	2,133	2,130
No. of rRNAs	19	19	19
No. of tRNAs	62	62	63
No. of other RNAs	4	4	4
No. of pseudogenes	100	101	100
Plasmids			
Plasmid name	pSH_1b_1	pSH_7b_1	pSH_12b_1
Accession no.	CP071513	CP071509	CP071506
Illumina short-read coverage	2,100×	1,700×	1,800×
Size (bp)	41,143	39,811	38,899
GC content (%)	31.7	31.0	30.4
No. of genes	44	44	44
Plasmid name	pSH_1b_2	pSH_7b_2	pSH_12b_2
Accession no.	CP071514	CP071510	CP071507
Illumina short-read coverage	14,600×	5,000×	18,900×
Size (bp)	5,922	13,654	3,856
GC content (%)	52.9	31.0	31.2
No. of genes	8	15	4
Plasmid name	pSH_1b_3	pSH_7b_3	
Accession no.	CP071515	CP071511	
Illumina short-read coverage	22,000×	17,800×	
Size (bp)	3,856	3,856	
GC content (%)	31.2	31.2	
No. of genes	4	4	

### Data availability.

The sequences have been deposited in the GenBank database under BioProject accession number PRJNA707584. The Sequence Read Archive (SRA) for the raw reads and the NCBI RefSeq accession numbers are provided in Table 1.
